# Gut microbiome changes in critically ill adults: a systematic review of longitudinal sequencing studies

**DOI:** 10.62675/2965-2774.20260392

**Published:** 2026-07-01

**Authors:** Christina-Chrysanthi Theocharidou, Zafeiris Tsinaris, Athanasia-Marina Peristeri, Olympia Akritidou, Anna Nikopoulou

**Affiliations:** 1 1st Intensive Care Unit General Hospital of Thessaloniki “G. Papanikolaou” Thessaloniki Greece 1st Intensive Care Unit, General Hospital of Thessaloniki “G. Papanikolaou” -Thessaloniki, Greece.; 2 Department of Microbiology AHEPA University Hospital Aristotle University of Thessaloniki Thessaloniki Greece Department of Microbiology, AHEPA University Hospital, Aristotle University of Thessaloniki - Thessaloniki, Greece.; 3 Department of Internal Medicine General Hospital of Thessaloniki “G. Papanikolaou” Thessaloniki Greece Department of Internal Medicine, General Hospital of Thessaloniki “G. Papanikolaou” - Thessaloniki, Greece.

**Keywords:** Critical illness, Dysbiosis, Gastrointestinal microbiome, Sequencing studies, Infection, Drug resistance, multiple, bacterial, Faecalibacterium, Critical care, Enterococcus, Anti-bacterial agents, Intensive care units

## Abstract

**Objective:**

Critical illness profoundly alters the gut microbiome, yet its temporal evolution and clinical relevance remain unclear. This systematic review aimed to synthesize evidence from longitudinal sequencing studies describing gut microbiome changes in critically ill adults and their association with clinical outcomes.

**Methods:**

We systematically searched MEDLINE®, Scopus, and Cochrane CENTRAL from inception to May 2025 for longitudinal observational studies analyzing gastrointestinal samples by sequencing in adult critically ill patients at ≥ 2 times points. Extracted data included study and patient characteristics, as well as microbiome outcomes, including alpha and beta diversity metrics and taxonomic abundance profiles. Due to heterogeneity, we undertook a structured descriptive synthesis: alpha diversity results were grouped by trajectory and compared across intensive care unit populations; beta diversity findings were tabulated and narratively synthesized; and reported associations with mortality and multidrug-resistant organism colonization were summarized narratively. Risk of bias was assessed with RoBANS 2, and certainty of evidence with GRADE.

**Results:**

Thirty-six studies comprising 2,067 critically ill adults were included. Most used 16S rRNA sequencing targeting the V4 region. A decline in alpha diversity was reported in 18 out of 31 studies, while 8 found no change and 4 mixed patterns. Beta diversity shifts over time were reported in 11 studies. Taxonomic analyses consistently revealed the expansion of opportunistic taxa such as *Enterococcus*, *Klebsiella*, and other *Enterobacteriaceae*, alongside the depletion of obligate anaerobes, including *Blautia*, *Coprococcus*, and *Faecalibacterium*. Early low diversity and pathogen-dominated microbiomes were associated with increased mortality. Associations with multidrug-resistant organism colonization were inconsistent. Certainty of evidence (GRADE) for all outcomes was rated very low due to heterogeneity and imprecision.

**Conclusion:**

Longitudinal sequencing studies demonstrate progressive loss of microbial diversity and enrichment of pathogenic taxa during critical illness. These shifts, particularly *Enterococcus* and *Klebsiella* overgrowth, correlate with adverse outcomes and may reflect the combined effects of antibiotics, disease severity, and critical care interventions. Standardized sampling, sequencing, and reporting protocols are needed to enable meta-analytic synthesis and guide microbiome-targeted interventions in the intensive care unit.

## INTRODUCTION

Critical illness exerts multiple pressures on the gut microbiome; antibiotics, altered nutrition, reduced motility, and immune dysfunction drive microbial shifts.^(1-4)^ The gut ecosystem is disrupted, leading to dysbiosis, characterized by loss of microbial diversity, expansion of pathobionts^(1,2,5)^ and increased clinical risk. The gut microbiome normally prevents overgrowth of antibiotic-resistant organisms.^(6)^ When its barrier is lost, the gastrointestinal tract can become a reservoir of potential pathogens, including multidrug-resistant *Enterococcus* and *Klebsiella*. These organisms translocate across the compromised gut barrier or seed distant sites, thereby increasing the risk of nosocomial infections.^(7,8)^ Antibiotic use, while life-saving, may accelerate loss of diversity and promote selective expansion of resistant taxa, creating a cycle of dysbiosis and infection risk within the intensive care unit (ICU) environment.^(9-11)^

Recent research has supported the notion that temporal loss of microbial diversity may be associated with nosocomial infections, sepsis, and mortality.^(4,12-14)^ Previous reviews^(15,16)^ have summarized cross-sectional or mixed designs, but none have systematically assessed changes across multiple time points. Traditional culture methods fail to capture the full extent of temporal shifts because many gut microbes are predominantly anaerobic and unculturable.^(17)^ Sequencing-based approaches, particularly 16S rRNA gene and shotgun metagenomics, have enabled a deeper understanding of gut microbial dynamics in the ICU setting.^(18,19)^ With this review, we attempt to address this gap by focusing exclusively on longitudinal sequencing studies.

This systematic review aims to synthesize evidence from longitudinal sequencing studies describing gut microbiome changes in critically ill adults and their association with clinical outcomes.

## METHODS

### Search strategy

This systematic review was prospectively registered with a publicly available protocol in PROSPERO (CRD420251044457) and conducted according to Preferred Reporting Items for Systematic reviews and Meta-Analyses (PRISMA) guidelines.^(20)^ A search was conducted across MEDLINE®/PubMed®, Scopus, and the Cochrane Central Register of Controlled Trials (CENTRAL) up to 26th May 2025. The search included combinations of keywords related to gut microbiome, critical illness, and sequencing methods. Full search strategies are detailed in table 1S (Supplementary Material). Reference lists in full-text reports were also screened.

### Inclusion criteria

- Population: studies recruiting adult patients (≥ 18 years) admitted to intensive care units were included.
- Study design: longitudinal, observational, non-randomized studies were included.
- Methods: only studies using sequencing methods in gastrointestinal samples (stool, rectal swabs, or gastric aspirates) were included.
- Sampling: studies with multiple successive sampling timepoints (≥ 2) during ICU stay were included.
- Language restrictions: only studies published in languages understood by the review team (English, French, German, Spanish, Italian, Dutch, Greek) were included.

### Exclusion criteria

- Population: studies with pediatric and perioperative patients with no defined ICU stay were excluded.
- Study design and type: studies without microbiome data, studies not using sequencing methods to assess the gut microbiome, and cross-sectional studies (studies with only one time point during the ICU stay) were excluded. Interventional studies, reviews, editorials, case reports, animal studies, *in vitro* studies, abstract-only studies, and conference abstracts were excluded.
- Sample size: studies with five patients or fewer were excluded.

### Screening, risk of bias, and data extraction

The “PICO Portal” platform was used for removing duplicates, screening abstracts, and full-text records.^(21)^ Two reviewers independently screened abstracts and full-text reports, while differences were resolved through consensus. Two reviewers independently performed data extraction and risk-of-bias assessment. Discrepancies were resolved by discussion. We extracted study characteristics (country, design, sample type, number and timing of samples, sequencing platform and target region, bioinformatic pipeline, diversity metrics), patient characteristics (age, ICU population, illness severity), baseline patient factors (antibiotic exposure, enteral nutrition, immunocompromised status, gastrointestinal comorbidities) and microbiome outcomes, including changes in alpha and beta diversity, shifts in taxonomic composition, and associations with mortality and multidrug-resistant organism (MDRO) colonization.

We used the “RoBANS 2 tool”^(22)^ for non-randomized studies to assess risk of bias across eight domains related to gut microbiome changes in diversity and pathogen abundance. Two reviewers independently evaluated the certainty of evidence using the Grading of Recommendations Assessment, Development and Evaluation (GRADE).^(23)^ Because all studies were observational cohorts, certainty ratings started at low.

### Analysis

A meta-analysis was not planned due to expected heterogeneity in study methods. We undertook a structured descriptive synthesis, tabulating study and patient characteristics, and outcomes. Alpha diversity results were grouped by trajectory and compared across ICU populations. Beta diversity findings were tabulated and narratively synthesized. Changes in abundances and reported associations with mortality and MDRO colonization were summarized narratively. An exploratory analysis compared study sizes across alpha-diversity trajectory groups.

Results are presented as mean ± standard deviation (SD) or median (interquartile range [IQR]), as appropriate. Statistical analyses were performed in IBM Statistical Package for the Social Sciences (SPSS) 26, with a level of significance at p < 0.05. Visualizations were created in Python, except for the phylogenetic tree, which was generated using the JavaScript library D3.js.

## RESULTS

### Study characteristics

**Figure 1:**
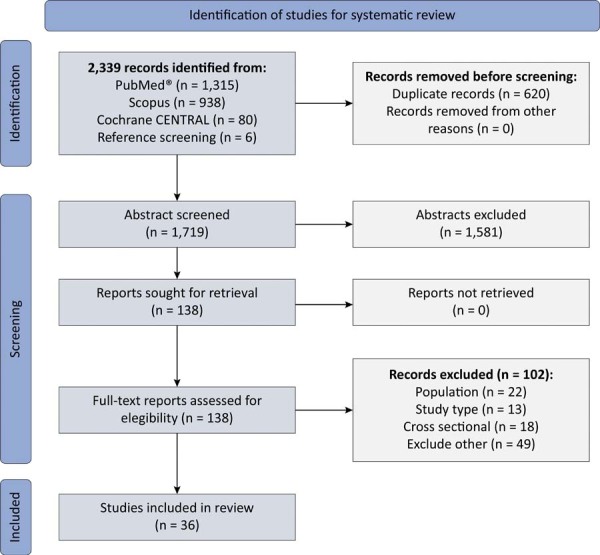
PRISMA flow diagram.

The screening process is shown in the PRISMA flowchart (Figure 1). One study^(24)^ retrospectively included the cohort from an earlier study;^(11)^ therefore, the two were treated as a single dataset. Another study^(25)^ was excluded because, despite its longitudinal design, only one sample was clearly obtained during the ICU stay. A study^(2)^ was excluded because fewer than five cases had undergone sequencing at multiple time points. Two studies, registered under the same European Nucleotide Archive (ENA) protocol, were included as separate entries due to distinct populations, different research centers, and non-overlapping time periods.^(4,26)^ One study was a retrospective study.^(27)^ We included 36 studies, encompassing 2,067 ICU patients (Table 1). The median study size was 39.5 patients (IQR 23.5 - 91.5), and the median number of samples analyzed was 114.5 (IQR 52.5 - 212). Patient cohorts mostly reported mean or median ages in the sixth to seventh decades, although age varied across clinical populations (Table 2S - Supplementary Material). Reporting of baseline host factors according to study criteria also varied (Table 3S - Supplementary Material). Patients with prior antibiotic exposure were excluded from eight studies. Nutrition exposure was reported in 12 studies, but enteral feeding was an inclusion criterion in 3 of them. Immunocompromised patients were excluded from 8 studies; 17 studies excluded patients with gastrointestinal comorbidities.

**Table 1:** Characteristics of longitudinal studies on gut microbiome changes in critically ill patients

Study	Country	Centers	Size (n)	Samples analyzed	Population	Population subgroups	Time points (n)
McDonald et al.^(1)^	United States, Canada	4	115	230	Critically ill	No subgroup	2
Park et al.^(^^3^^)^	United States	1	90	340	Sepsis	VRE positive/VRE negative	5
Agudelo-Ochoa et al.^(^^4^^)^	Colombia	5	155	250	Critically ill	Sepsis/no sepsis	3
Ravi et al.^(^^10^^)^	United Kingdom	1	24	236	Long stay critically ill	No subgroup	> 2
Livanos et al.^(^^11^^)^	United States	5	93	184	Critically ill	No subgroup	2
Wozniak et al.^(^^12^^)^	Switzerland	1	96	192	Critically ill with APACHE ≥ 20 or SAPS ≥ 38	Survivors/non-survivors	2
Saikrishna et al.^(^^17^^)^	India	1	35	86	Critically ill with enteral feeding tolerance and APACHE II ≤ 25	Probiotics/no probiotics	3
Jaillier-Ramirez et al.^(^^26^^)^	Colombia	3	28	56	Sepsis	No subgroup	2
Liu et al.^(^^27^^)^	China	1	14	40	Sepsis	Sepsis non-severe/sepsis severe	3
Kritikos et al.^(^^28^^)^	Switzerland	1	38	93	Critically ill	Pneumonia/other infection/not infected	5
Cuenca et al.^(^^29^^)^	Spain	1	98	169	Critically ill	COVID-19/other	3
Nielsen et al. ^(^^30^^)^	Denmark	1	34	78	Neurocritically ill	Broad antibiotics/narrow antibiotics	2
Wang et al. ^(^^31^^)^	China	1	29	42	Critically ill	*Clostridioides difficile* positive/ negative	>2
Wang et al. ^(^^32^^)^	China	1	7	42	Severe burns	No subgroup	6
Zhou et al.^(^^33^^)^	China	1	10	20	Sepsis	No subgroup	2
Magnan et al.^(^^34^^)^	France	1	60	120	Septic shock	No subgroup	2
Ojima et al.^(^^35^^)^	Japan	1	71	238	Critically ill	No subgroup	>4
Ojima et al.^(^^36^^)^	Japan	1	12	48	Critically ill	No subgroup	4
Xu et al.^(^^37^^)^	China	1	24	48	Sepsis	Enteral nutrition tolerance/ enteral nutrition intolerance	2
Xu et al.^(^^38^^)^	China	1	98	206	Neurocritically ill	No subgroup	10
Long et al.^(^^39^^)^	China	1	23	109	Critically ill	Sepsis/ No sepsis	3
Howard et al.^(^^40^^)^	United States	1	12	24	Critically ill trauma	No subgroup	3
Kitsios et al.^(^^41^^)^	United States	1	216	349	Acute respiratory failure	No subgroup	3
Zhou et al.^(^^42^^)^	China	1	62	124	Critically ill without pulmonary or abdominal infection	Survivors/non-survivors	2
Luan et al.^(^^43^^)^	China	2	41	70	Sepsis	Survivors/non-survivors	2
Mu et al.^(^^44^^)^	China	1	77	312	Critically ill	Sepsis/no sepsis	2
Yang et al.^(^^45^^)^	China	1	20	49	Critically ill	Sepsis/no sepsis	3
Pettigrew et al.^(^^46^^)^	United States	1	109	218	Critically ill	CRPA colonization/no colonization/ No antibiotics and no colonization	> 2
Fontaine et al.^(^^47^^)^	France	1	31	48	Critically ill	No subgroup	> 2
Patrier et al.^(^^48^^)^	France	1	95	379	COVID-19 critically ill	No subgroup	> 2
Garcia et al.^(^^49^^)^	Spain	1	62	172	Liver disease/liver transplantation	No subgroup	> 2
Schlechte et al.^(^^50^^)^	Canada	1	51	124	Critically ill	No subgroup	3
Bansal et al.^(^^55^^)^	Canada	1	9	70	Critically ill	No subgroup	7
Kuo et al.^(^^56^^)^	United States	1	78	156	Critically ill	Low 3-indoxyl sulfate/ high 3-indoxyl sulfate	2
Chernevskaya et al.^(^^57^^)^	Russia	2	18	94	Critically ill	Acute critically ill/ chronically critically ill	3
Yeh et al.^(^^58^^)^	United States	1	32	79	Critically ill trauma	No subgroup	3

VRE - vancomycin-resistant *Enterococcus*; APACHE - Acute Physiology and Chronic Health Evaluation; SAPS - Simplified Acute Physiology Score; CRPA - carbapenem-resistant *Pseudomonas aeruginosa*.

Characteristics of sequencing methodologies are summarized in table 2. Among 16S rRNA sequencing studies, the V4 region was most frequently targeted (30 studies), followed by V3 (Figure 1S - Supplementary Material). Choice and use of diversity metrics were heterogeneous (Table 4S - Supplementary Material). Alpha diversity was calculated in 34 studies, with the Shannon index most frequently employed (27 studies), followed by Chao1 (15 studies) and the Simpson index (13 studies). Beta diversity analysis was performed in 23 studies, with Bray-Curtis dissimilarity (13 studies) and UniFrac distances (13 studies) predominantly used.

**Table 2:** Characteristics of sequencing methods

Study	Sample type	Sequencing type	Sequencing platform	Region sequenced*	Sequences public	Sequencing clustering method	Reference database
McDonald et al.^(^^1^^)^	Stool	16S rRNA	Illumina MiSeq	V4	Yes	OTU	Greengenes
Park et al.^(^^3^^)^	Rectal swab	16S rRNA	Illumina MiSeq	V3-V4	Yes	NA	SILVA
Agudelo-Ochoa et al.^(^^4^^)^	Perirectal swab	16S rRNA	Illumina MiSeq	V3-V4	Yes	OTU	SILVA
Ravi et al.^(^^10^^)^	Stool	Shotgun metagenomic	Illumina NextSeq	NI	Yes	MAGs	De novo
Livanos et al.^(^^11^^)^	Rectal swab	16S rRNA	Illumina HiSeq	V4	Yes	OTU	Greengenes
Wozniak et al.^(^^12^^)^	Stool	16S rRNA	PacBio Sequel IIe	V1-V9	Yes	ASV	EzBioCloud
Saikrishna et al.^(^^17^^)^	Stool	16S rRNA	Illumina MiSeq, Nanopore	NA	Yes	ASV	SILVA
Jaillier-Ramirez et al.^(^^26^^)^	Rectal swab	16S rRNA	Illumina MiSeq	V3-V4	Yes	ASV	SILVA
Liu et al.^(^^27^^)^	Rectal swab, Stool	16S rRNA	Illumina MiSeq	V3-V4	No	OTU	NA
Kritikos et al.^(^^28^^)^	Rectal swab	16S rRNA	Illumina MiSeq	V3-V4	Yes	ASV	EzBioCloud
Cuenca et al.^(^^29^^)^	Rectal swab	16S rRNA	Illumina MiSeq	V4	Yes	ASV	Greengenes
Nielsen et al.^(^^30^^)^	Rectal swab	Shotgun metagenomic	Illumina Novaseq	NI	Yes	MGS	Clinical Microbiomics HGMGS
Wang et al.^(^^31^^)^	Stool	16S rRNA	Illumina MiSeq	V3-V4	No	OTU	SILVA
Wang et al.^(^^32^^)^	Stool	16S rRNA	Illumina MiSeq	V4	No	OTU	Greengenes
Zhou et al.^(^^33^^)^	Stool	16S rRNA	Illumina MiSeq	V3-V4	No	OTU	NA
Magnan et al.^(^^34^^)^	Stool	16S rRNA	Illumina MiSeq	V3-V4	Yes	OTU	Greengenes
Ojima et al.^(^^35^^)^	Rectal swab	16S rRNA	Illumina MiSeq	V1-V2	No	OTU	Greengenes
Ojima et al.^(^^36^^)^	Rectal swab	16S rRNA	Illumina HiSeq	V4	No	OTU	Greengenes
Xu et al.^(^^37^^)^	Stool	16S rRNA	Illumina MiSeq/HiSeq	NA	No	OTU	NA
Xu et al.^(^^38^^)^	Stool	16S rRNA	Illumina Hiseq	V4	No	OTU	Greengenes
Long et al.^(^^39^^)^	Stool	16S rRNA	Illumina Novaseq	V4-V5	Yes	OTU	SILVA
Howard et al.^(^^40^^)^	Stool	16S rRNA	Illumina MiSeq	V4	No	OTU	Greengenes
Kitsios et al.^(^^41^^)^	Rectal swab, Stool	16S rRNA	Illumina MiSeq	V3-V4	Yes	ASV	Unite
Zhou et al.^(^^42^^)^	Stool	16S rRNA	Illumina MiSeq	V3-V4	Yes	OTU	NA
Luan et al.^(^^43^^)^	Rectal swab	16S rRNA	Illumina MiSeq	NA	No	OTU	NA
Mu et al.^(^^44^^)^†	Stool	16S rRNA^b^	Illumina MiSeq, Illumina X10	V3-V4	Yes	OTU	SILVA (alignment), RDP (assignment)
Yang et al.^(^^45^^)^	Stool	16S rRNA	Illumina MiSeq	V3-V4	Yes	OTU	SILVA
Pettigrew et al.^(^^46^^)^	Perirectal swab	16S rRNA	Illumina MiSeq	V4	No	OTU	NA
Fontaine et al.^(^^47^^)^	Stool	16S rRNA	Illumina MiSeq	V4	Yes	OTU	NA
Patrier et al.^(^^48^^)^	Rectal swab	16S rRNA	Illumina MiSeq	V3-V4	Yes	OTU	NA
Garcia et al.^(^^49^^)^	Rectal swab	16S rRNA	Illumina MiSeq	V3-V4	No	ASV	SILVA
Schlechte et al.^(^^50^^)^	Rectal swab	16S rRNA	Illumina MiSeq	V4	Yes	ASV	SILVA
Bansal et al.^(^^55^^)^	Rectal swab, Stool	16S rRNA	Illumina MiSeq	V4	Yes	OTU	RDP
Kuo et al.^(^^56^^)^	Rectal swab	16S rRNA	Illumina MiSeq	V4	No	ASV	Greengenes
Chernevskaya et al.^(^^57^^)^	Rectal swab	16S rRNA	Ion Torrent	V2-V4, V6-V9	Yes	OTU	Greengenes
Yeh et al.^(^^58^^)^	Rectal swab, Stool	16S rRNA	Illumina HiSeq	V4	No	OTU	Greengenes

OTU - operational taxonomic unit; NA - not available; NI - not indicated; MAGs - metagenomic assembly genomes; ASV - amplicon sequence variant; MGS - Metagenomic Species, HGMGS - human gut metagenomic species; RDP - ribosomal database project. * Applied to amplicon sequencing studies; † Mu et al. also performed whole genome sequencing for *Klebsiella pneumoniae* and *Enterococcus faecium*.

### Risk of bias

**Figure 2:**
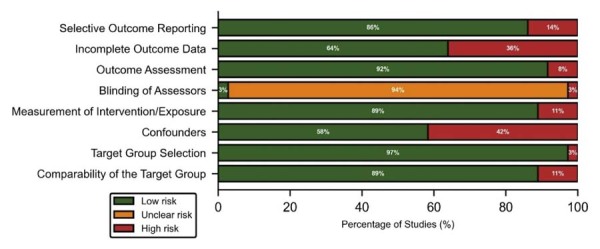
Summary of risk of bias assessment (RoBANS-2) in gut microbiome studies in critically ill patients.

Risk of bias assessment across studies is presented in figure 2. Study-specific risk of bias assessment is shown in figure 2S (Supplementary Material). Overall, methodological quality was acceptable across most domains, except for assessor blinding, which was mostly unclear but expected given the studies’ design.

### Gut microbiome diversity

Alpha diversity metrics at different time points were reported in 31 of 36 studies (Table 5S - Supplementary Material). A decrease in alpha diversity during ICU stay was observed in 18 studies. In a subgroup of patients without infection who did not receive antibiotics, the alpha diversity did not change over time.^(28)^ No change in diversity was described in eight studies. Mixed patterns were reported in four studies. In a small study of patients with severe burn, alpha diversity showed recovery after 5 weeks.^(32)^ Only one study^(33)^ observed an increase in alpha diversity in sepsis patients between admission and 72 hours. When studies were grouped by clinical context (Table 6S - Supplementary Material), alpha diversity decreased in 12 out of 22 general/mixed ICU cohorts and in 4 out of 7 sepsis cohorts. The 8 studies reporting no change in alpha diversity were distributed across general/mixed ICUs (n = 4), trauma or burns (n = 2), and sepsis (n = 2) populations, and were not restricted to a specific subgroup.

We examined whether study size varied by reported alpha diversity changes. The single study that observed an increase in alpha diversity was excluded. Among the remaining 30 studies, the median study size was 74 [IQR 28 - 100.5] in studies reporting decreased diversity (n = 18), 31.5 [IQR 22 - 38] in those reporting no change (n = 8), and 31.5 [IQR 26.5 - 66] in those reporting mixed patterns (n = 4). A test comparing these three groups did not reach statistical significance (p = 0.21).

Beta diversity was evaluated in 23 studies, 11 of which examined temporal change during ICU stay. Among these, ten studies reported significant temporal changes in beta diversity in longitudinal samples.^(1,4,26,28,29,32,34,35,37,38)^ One study in neurocritically ill patients found no temporal changes in beta diversity.^(30)^

### Taxonomic composition and abundance patterns

**Figure 3:**
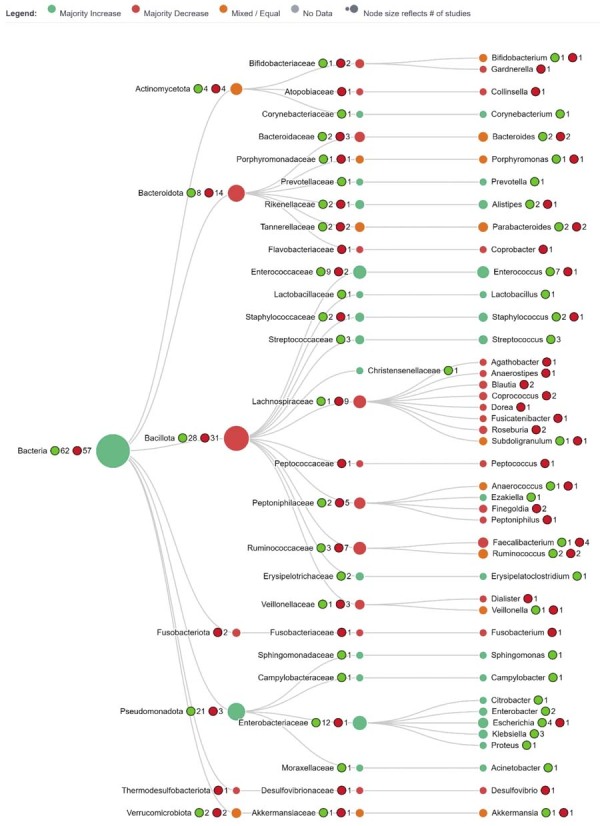
Phylogenetic tree summarizing reported temporal shifts in abundance across studies at the phylum, family, and genus levels (node color indicates study majority consensus on abundance: green for increased, red for decreased, orange for mixed results, node size is proportional to the number of supporting studies, numbers next to nodes refer to number of studies).

Temporal shifts in gut microbial taxonomic composition were reported in 33 of 36 studies. A phylogenetic tree illustrates the pattern of reported increases, decreases, and mixed results for each taxonomic level, by study majority consensus (Figure 3). An interactive HTML version of the phylogenetic tree is available online at osf.io/pn4me. Detailed abundance changes are presented in table 7S (Supplementary Material).

### Increased abundance over time

An increase in the relative abundance of taxa was reported in 17 of 33 studies that evaluated taxonomic change. *Enterococcus* was among the most frequently reported enriched taxa, with increasing abundance during ICU stay in nine studies.^(10,11,27,29,32,33,37,39,40)^ *Streptococcus*^(10,17,33)^ and *Staphylococcus*^(1,41)^ were also enriched during ICU stay, in three and two studies, respectively. Two studies reported an increase in Bacillota, a change largely driven by expansion of *Enterococcaceae* rather than a uniform rise across the phylum.^(32,42)^ Species from the *Pseudomonadota phylum* were increased in five studies.^(1,26,29,37,43)^ An increase in the abundance of *Enterobacteriaceae*, including *Klebsiella*, *Enterobacter*, *Escherichia,* and *Proteus,* was reported in five studies.^(10,29,33,43,44)^ Among them, *Escherichia* and *Klebsiella* were the most frequently increased genera, with the latter becoming the dominant genus in the first week of ICU stay in one cohort.^(44)^

### Decreased abundance over time

A reduction in the relative abundance of taxa was reported in 4 of 33 studies that investigated taxonomic change. Members of the Bacillota, apart from *Enterococcus*, *Staphylococcus*, and *Streptococcus*, showed reduced relative abundance.^(1,11,33)^ Decreased abundance was reported for the genera *Blautia*, *Coprococcus*, and the majority of *Lachnospiraceae*.^(11,41)^ Mixed results were reported for Bacteroidota in three studies^(28,37,42)^ and for *Parabacteroides* and *Coprobacter* in one study.^(12)^ Anaerobic-spectrum antibiotic use was associated with progressive declines in obligate anaerobes and a corresponding increase in potentially pathogenic taxa, indicating antibiotic-driven compositional shifts in one study.^(41)^

### *Enterococcus* abundance

*Enterococcus* abundance was examined in 11 studies. *Enterococcus* was identified as dominant or expanding, with levels peaking between days 3 and 7 of ICU stay in six studies.^(11,27,29,32,34,40)^ Higher abundances were observed in sepsis *versus* non-sepsis patients in one study,^(45)^ and in those receiving piperacillin-tazobactam in another.^(46)^ Patients with sepsis who were colonized with vancomycin-resistant *Enterococcus* (VRE) at ICU admission exhibited persistently reduced alpha diversity and increased relative abundance of *Enterococcus*, with further elevations observed during vancomycin treatment.^(3)^ Similarly, colonization with *Enterococcus faecium* was associated with decreased microbial diversity.^(47)^ Elevated *Enterococcus* abundance correlated with lower microbial diversity and a higher risk of mortality.^(48)^

### Sepsis

Among studies recruiting patients with sepsis, a decline in alpha diversity over time was reported in four studies,^(3,26,27,34)^ while no change was observed in two^(37,43)^ and an increase in one.^(33)^ The taxa most frequently reported as enriched were *Enterococcus*^(27,33,37)^ and *Pseudomonadota*.^(26,37,43)^ Increased abundances of *Streptococcus*^(33)^ and *Enterobacteriaceae*^(43)^ were also reported. Reductions in Bacillota (excluding *Enterococcus*, *Staphylococcus*, and *Streptococcus*) were reported in one study,^(33)^ while mixed changes in Bacteroidota were described in another.^(37)^

Differences in gut microbiome composition between sepsis and non-sepsis patients were reported in three studies. Non-sepsis patients exhibited higher abundances of *Fusobacterium*, *Anaerococcus*, *Peptostreptococcus*, *Granulicatella*, *Streptococcus*, *Prevotella*, and *Megasphaera elsdenii* compared to those with sepsis in two studies.^(4,45)^ In one study, *Enterococcus* was more abundant in sepsis patients.^(45)^ Furthermore, in sepsis, enrichment of *Stenotrophomonas*, *Pseudaminobacter*, *Pseudonocardia*, and *Lactobacillus*, and decreased *Faecalibacterium*, *Ruminococcus*, *Eubacterium*, and *Collinsella* was reported in one study.^(39)^

### Association between changes in microbiome and multidrug-resistant organisms

Four studies investigated the relationship between gut microbiome and the presence or dynamics of MDROs in critically ill populations. In one study, loss of gut microbial diversity and higher abundances of taxa now classified within the family *Peptoniphilaceae* (formerly referred to as ‘Clostridiales Family XI *incertae sedis*’) and *Prevotellaceae* were associated with protection from MDROs, whereas increased *Enterobacteriaceae* abundance was associated with a higher risk of MDRO acquisition.^(49)^ In a study with neurocritically ill patients, no significant differences were found in the colonization of extended-spectrum beta-lactamase-producing organisms, vancomycin-resistant genes, or carbapenemase producers based on narrow or broad antibiotic exposure.^(30)^ Another study found no link between the intestinal relative abundance of multidrug-resistant Gram-negative bacteria and microbial richness or diversity.^(47)^ A metagenomic analysis in long-stay ICU patients identified resistance gene carriage but did not specify direct clinical associations.^(10)^

### Mortality

Nine studies investigated changes in the gut microbiome and mortality.^(4,12,34,38,42,43,48-50)^ Lower alpha diversity early in ICU stay was associated with increased risk of death.^(12,50)^ Alterations in microbial composition between survivors and non-survivors were observed, including temporal shifts in community structure^(43)^ and enrichment of taxa such as *Christensenellaceae* and *Erysipelotrichaceae* in non-survivors.^(38)^ An increase in the abundance of *Enterobacteriaceae* was associated with death within 180 days in one study.^(38)^ Samples in deceased patients were more likely to show increased abundances of potentially pathogenic taxa, including *Enterococcus*, *Staphylococcus aureus*, *Pseudomonas*, *Clostridiaceae*, and *Salmonella*.^(4,34,48)^ *Enterococcaceae* were independently associated with 28-day mortality in one study.^(42)^ Serial increases in *Enterococcus* were linked to poor outcomes in COVID-19 patients.^(48)^ Higher diversity and abundance of *Peptoniphilaceae* and *Prevotellaceae* were associated with a lower risk of death.^(49)^ Regarding mortality analyses in these studies, adjustment for age was inconsistent. Only two studies explicitly included age in multivariable models,^(38,48)^ one study evaluated age during feature selection. However, it did not retain it in the final model,^(49)^ and the remaining studies did not adjust for age.

### Certainty of evidence (GRADE)

Certainty ratings for three outcomes are summarized in table 3. Evidence was rated very low due to inconsistent effect direction and imprecision concerns. No upgrading factors were applied to any outcome.

**Table 3:** Certainty of evidence in three outcomes (GRADE)

Outcome	Overall certainty	Symbol
Progressive decline in gut-microbiome diversity during ICU stay	Very low	⊕◯◯◯
Higher mortality when there is early low diversity or microbiome is pathogen-dominated	Very low	⊕◯◯◯
Increased abundance of *Enterococcus* after ICU admission	Very low	⊕◯◯◯

## DISCUSSION

This systematic review has several strengths. To our knowledge, it provides the largest synthesis of longitudinal gut microbiome data in critically ill adults, incorporating 36 sequencing-based studies and 2,067 patients. By focusing exclusively on longitudinal designs, it describes how the gut microbiome shifts after ICU admission. While only 16S rRNA and metagenomic sequencing studies are included, detailed reporting of sample types, sequencing platforms, and analytic pipelines provides context for interpreting heterogeneity.

Across studies, two patterns emerged: a progressive loss of microbial diversity and an overgrowth of opportunistic taxa, especially *Enterococcus* and *Enterobacteriaceae* such as *Klebsiella*. These shifts are clinically relevant and align with adverse outcomes reported in critically ill populations, including sepsis, multidrug-resistant colonization, and mortality. Residual confounding by age, which affects both mortality and gut microbial diversity,^(51)^ cannot be ruled out when interpreting associations between changes in gut microbiome and mortality.

The observed loss of diversity and expansion of opportunistic taxa likely reflect shared features of critical illness and its management. Broad-spectrum antibiotic exposure favors dominance of organisms such as *Enterococcus* and *Enterobacteriaceae*, while physiological stress, impaired gut barrier function, and altered host immunity further destabilize microbial communities.^(52,53)^ Disruption of enteral nutrition may also contribute to unfavorable shifts in microbial community composition.^(54)^ Together, these factors provide a plausible biological context for the recurrent microbiome patterns reported across studies. However, causal relationships cannot be inferred from the available observational data, as different studies employed variable inclusion and exclusion criteria.

A previous systematic review of 26 studies found no clear association between decreased gut microbiome diversity and mortality.^(15)^ While their meta-analysis using the Shannon index qualitatively as a binary factor provides quantitative estimates, it was constrained by the limited availability of extractable data from four studies. Another systematic review of 13 studies reported variable trajectory patterns of microbiome disruption in the ICU, but only in patients with persistent critical illness.^(16)^ The researchers did not carry out the planned meta-analysis due to the lack of available primary outcome data and study heterogeneity. Likewise, we deliberately avoided meta-analysis, as pooling across variable sequencing platforms, time points, and diversity metrics would risk misleading conclusions. A descriptive synthesis more accurately reflects the available evidence and highlights where methodological standardization is most needed.

There are also methodological limitations specific to this review that affect the certainty of the evidence. While adhering to PRISMA guidelines, we performed only a descriptive synthesis of the evidence. We did not extract numeric data from plots using digitalization tools, which may have led to the omission of otherwise usable but unverified data. Lastly, although our decision to avoid meta-analysis was methodologically sound, it limits the ability to generate pooled effect estimates for diversity change or mortality associations.

Several factors likely explain variability across studies. First, heterogeneity can be attributed to inconsistent use of population inclusion and exclusion criteria, particularly antibiotic exposure, nutrition requirements, immunocompromised status, and gastrointestinal comorbidities. Second, sampling methods varied (stool, rectal, or perirectal swabs), each with differing microbial load yield and contamination risk.^(55)^ Moreover, most studies used only two or three unequally spaced time points, limiting insight into later phase dynamics or recovery patterns. Furthermore, different sequencing platforms exhibit distinct error profiles and read lengths, which influence raw data quality and the accuracy of taxonomic assignments. Differences in the hypervariable regions used, as shown in table 2 and figure 1S (Supplementary Material), present a key limitation, since each region yields distinct taxonomic resolution and biases.

Finally, the use of heterogeneous diversity metrics complicates interpretation. Alpha diversity indices emphasize different properties of the microbial community: Chao1 reflects richness, Shannon combines richness and evenness, and Simpson weights dominance by abundant taxa. Consequently, microbiome changes characterized by loss of evenness and overgrowth of dominant taxa may be detected by Shannon or Simpson indices but not by richness-based metrics. Similarly, differences in beta diversity measures (e.g., Bray-Curtis *versus* UniFrac) further limit cross-study comparability. Although a few consistent biological signals emerged, the overall certainty of evidence remains very low. Consequently, our findings should be interpreted as hypothesis-generating rather than definitive.

Future longitudinal gut microbiome studies in critical care research should adopt standardized sampling protocols, concordant sequencing technologies, and transparent reporting of analytic pipelines. They should harmonize inclusion and exclusion criteria to assess antibiotic exposure at inclusion when feasible (e.g., in antibiotic-naïve cohorts) and ensure explicit reporting of enteral nutrition and immune status. This research field would benefit from the development of a universal reporting guideline specifically tailored to sequencing-based microbiome studies in critical illness. Establishing this foundation would reduce methodological heterogeneity, enhance reproducibility, and strengthen future evidence synthesis. Standardizing the full pipeline, from sample handling to phylogenetic placement, will be critical.

## CONCLUSION

Gut microbiome disruption is common in critical illness, characterized by declining diversity and expansion of taxa such as *Enterococcus* and *Klebsiella*. These changes are associated with poor outcomes. While current evidence remains heterogeneous, understanding and eventually modulating the gut microbiome may offer new avenues to improve outcomes in the intensive care unit.

## References

[1] McDonald D, Ackermann G, Khailova L, Baird C, Heyland D, Kozar R (2016). Extreme dysbiosis of the microbiome in critical illness. MSphere.

[2] Zaborin A, Smith D, Garfield K, Quensen J, Shakhsheer B, Kade M (2014). Membership and behavior of ultra-low-diversity pathogen communities present in the gut of humans during prolonged critical illness. MBio.

[3] Park H, Abrams JA, Uhlemann AC, Freedberg DE (2025). Gut colonization with vancomycin-resistant Enterococcus shapes the gut microbiome in the intensive care unit. J Infect Dis.

[4] Agudelo-Ochoa GM, Valdés-Duque BE, Giraldo-Giraldo NA, Jaillier-Ramírez AM, Giraldo-Villa A, Acevedo-Castaño I (2020). Gut microbiota profiles in critically ill patients, potential biomarkers and risk variables for sepsis. Gut Microbes.

[5] Shimizu K, Ogura H, Goto M, Asahara T, Nomoto K, Morotomi M (2006). Altered gut flora and environment in patients with severe SIRS. J Trauma.

[6] Horrocks V, King OG, Yip AY, Marques IM, McDonald JA (2023). Role of the gut microbiota in nutrient competition and protection against intestinal pathogen colonization. Microbiology (Reading).

[7] Nagpal R, Yadav H (2017). Bacterial Translocation from the gut to the distant organs: an overview. Ann Nutr Metab.

[8] Donskey CJ (2004). The role of the intestinal tract as a reservoir and source for transmission of nosocomial pathogens. Clin Infect Dis.

[9] Wischmeyer PE, McDonald D, Knight R (2016). Role of the microbiome, probiotics, and 'dysbiosis therapy' in critical illness. Curr Opin Crit Care.

[10] Ravi A, Halstead FD, Bamford A, Casey A, Thomson NM, van Schaik W (2019). Loss of microbial diversity and pathogen domination of the gut microbiota in critically ill patients. Microb Genom.

[11] Livanos AE, Snider EJ, Whittier S, Chong DH, Wang TC, Abrams JA (2018). Rapid gastrointestinal loss of Clostridial Clusters IV and XIVa in the ICU associates with an expansion of gut pathogens. PLoS One.

[12] Wozniak H, Gaïa N, Lazarevic V, Le Terrier C, Beckmann TS, Balzani E, Gut Microbiota working group (2024). Early reduction in gut microbiota diversity in critically ill patients is associated with mortality. Ann Intensive Care.

[13] Tamburini FB, Andermann TM, Tkachenko E, Senchyna F, Banaei N, Bhatt AS (2018). Precision identification of diverse bloodstream pathogens in the gut microbiome. Nat Med.

[14] Freedberg DE, Zhou MJ, Cohen ME, Annavajhala MK, Khan S, Moscoso DI (2018). Pathogen colonization of the gastrointestinal microbiome at intensive care unit admission and risk for subsequent death or infection. Intensive Care Med.

[15] Evans T, Ali U, Anderton R, Raby E, Manning L, Litton E (2023). Lower gut dysbiosis and mortality in acute critical illness: a systematic review and meta-analysis. Intensive Care Med Exp.

[16] Tang E, Doan N, Evans T, Litton E (2024). Lower gastrointestinal tract dysbiosis in persistent critical illness: a systematic review. J Med Microbiol.

[17] Saikrishna K, Talukdar D, Das S, Bakshi S, Chakravarti P, Jana P (2023). Study on effects of probiotics on gut microbiome and clinical course in patients with critical care illnesses. Microb Ecol.

[18] Dickson RP (2016). The microbiome and critical illness. Lancet Respir Med.

[19] Clemente JC, Ursell LK, Parfrey LW, Knight R (2012). The impact of the gut microbiota on human health: an integrative view. Cell.

[20] Page MJ, McKenzie JE, Bossuyt PM, Boutron I, Hoffmann TC, Mulrow CD (2021). The PRISMA 2020 statement: an updated guideline for reporting systematic reviews. BMJ.

[21] Pico Portal (c2026). St. Petersburg FL.

[22] Seo HJ, Kim SY, Lee YJ, Park JE (2023). RoBANS 2: a revised risk of bias assessment tool for nonrandomized studies of interventions. Korean J Fam Med.

[23] Guyatt GH, Oxman AD, Vist GE, Kunz R, Falck-Ytter Y, Alonso-Coello P, GRADE Working Group (2008). GRADE: an emerging consensus on rating quality of evidence and strength of recommendations. BMJ.

[24] Fu Y, Moscoso DI, Porter J, Krishnareddy S, Abrams JA, Seres D (2020). Relationship between dietary fiber intake and short-chain fatty acid-producing bacteria during critical illness: a prospective cohort study. JPEN J Parenter Enteral Nutr.

[25] Aardema H, Lisotto P, Kurilshikov A, Diepeveen JR, Friedrich AW, Sinha B (2020). Marked changes in gut microbiota in cardio-surgical intensive care patients: a longitudinal cohort study. Front Cell Infect Microbiol.

[26] Jaillier-Ramírez AM, Valdés-Duque BE, Giraldo-Giraldo NA, Mesa V, Barbosa-Barbosa J, Yepes-Molina M (2022). Cambios en la microbiota intestinal de pacientes críticos con sepsis una semana después del ingreso a la Unidad de Cuidados Intensivos. Acta Colomb Cuid Intensivo.

[27] Liu Y, Guo Y, Hu S, Wang Y, Zhang L, Yu L (2023). Analysis of the dynamic changes in gut microbiota in patients with different severity in sepsis. BMC Infect Dis.

[28] Kritikos A, Bernasconi E, Choi Y, Scherz V, Pagani JL, Greub G (2025). Lung and gut microbiota profiling in intensive care unit patients: a prospective pilot study. BMC Infect Dis.

[29] Cuenca S, Soler Z, Serrano-Gómez G, Xie Z, Barquinero J, Roca J (2022). Dysbiosis: an indicator of COVID-19 severity in critically ill patients. Int J Mol Sci.

[30] Nielsen KL, Olsen MH, Pallejá A, Ebdrup SR, Sørensen N, Lukjancenko O (2021). Microbiome compositions and resistome levels after antibiotic treatment of critically ill patients: an observational cohort study. Microorganisms.

[31] Wang D, Dong D, Wang C, Cui Y, Jiang C, Ni Q (2020). Risk factors and intestinal microbiota: clostridioides difficile infection in patients receiving enteral nutrition at intensive care units. Crit Care.

[32] Wang X, Yang J, Tian F, Zhang L, Lei Q, Jiang T (2017). Gut microbiota trajectory in patients with severe burn: a time series study. J Crit Care.

[33] Zhou Y, Luo Y, Wang X, Luan F, Peng Y, Li Y (2023). Early gut microbiological changes and metabolomic changes in patients with sepsis: a preliminary study. Int Microbiol.

[34] Magnan C, Lancry T, Salipante F, Trusson R, Dunyach-Remy C, Roger C (2023). Role of gut microbiota and bacterial translocation in acute intestinal injury and mortality in patients admitted in ICU for septic shock. Front Cell Infect Microbiol.

[35] Ojima M, Shimizu K, Motooka D, Ishihara T, Nakamura S, Shintani A (2022). Gut dysbiosis associated with antibiotics and disease severity and its relation to mortality in critically ill patients. Dig Dis Sci.

[36] Ojima M, Motooka D, Shimizu K, Gotoh K, Shintani A, Yoshiya K, Nakamura S, Ogura H, Iida T, Shimazu T (2016). Metagenomic Analysis Reveals Dynamic Changes of Whole Gut Microbiota in the Acute Phase of Intensive Care Unit Patients. Dig Dis Sci.

[37] Xu W, Zhong M, Pan T, Qu H, Chen E (2022). Gut Microbiota and enteral nutrition tolerance in non-abdominal infection septic ICU patients: an observational study. Nutrients.

[38] Xu R, Tan C, Zhu J, Zeng X, Gao X, Wu Q (2019). Dysbiosis of the intestinal microbiota in neurocritically ill patients and the risk for death. Crit Care.

[39] Long X, Mu S, Zhang J, Xiang H, Wei W, Sun J (2023). Global signatures of the microbiome and metabolome during hospitalization of septic patients. Shock.

[40] Howard BM, Kornblith LZ, Christie SA, Conroy AS, Nelson MF, Campion EM (2017). Characterizing the gut microbiome in trauma: significant changes in microbial diversity occur early after severe injury. Trauma Surg Acute Care Open.

[41] Kitsios GD, Sayed K, Fitch A, Yang H, Britton N, Shah F (2024). Longitudinal multicompartment characterization of host-microbiota interactions in patients with acute respiratory failure. Nat Commun.

[42] Zhou P, Zou Z, Wu W, Zhang H, Wang S, Tu X (2023). The gut-lung axis in critical illness: microbiome composition as a predictor of mortality at day 28 in mechanically ventilated patients. BMC Microbiol.

[43] Luan F, Zhou Y, Ma X, Li Y, Peng Y, Jia X (2024). Gut microbiota composition and changes in patients with sepsis: potential markers for predicting survival. BMC Microbiol.

[44] Mu S, Xiang H, Wang Y, Wei W, Long X, Han Y (2022). The pathogens of secondary infection in septic patients share a similar genotype to those that predominate in the gut. Crit Care.

[45] Yang XJ, Liu D, Ren HY, Zhang XY, Zhang J, Yang XJ (2021). Effects of sepsis and its treatment measures on intestinal flora structure in critical care patients. World J Gastroenterol.

[46] Pettigrew MM, Gent JF, Kong Y, Halpin AL, Pineles L, Harris AD (2019). Gastrointestinal microbiota disruption and risk of colonization with carbapenem-resistant Pseudomonas aeruginosa in intensive care unit patients. Clin Infect Dis.

[47] Fontaine C, Armand-Lefèvre L, Magnan M, Nazimoudine A, Timsit JF, Ruppé E (2020). Relationship between the composition of the intestinal microbiota and the tracheal and intestinal colonization by opportunistic pathogens in intensive care patients. PLoS One.

[48] Patrier J, Villageois-Tran K, Szychowiak P, Ruckly S, Gschwind R, Wicky PH, French COVID Cohort Study Group (2022). Oropharyngeal and intestinal concentrations of opportunistic pathogens are independently associated with death of SARS-CoV-2 critically ill adults. Crit Care.

[49] Garcia ER, Vergara A, Aziz F, Narváez S, Cuesta G, Hernández M (2022). Changes in the gut microbiota and risk of colonization by multidrug-resistant bacteria, infection, and death in critical care patients. Clin Microbiol Infect.

[50] Schlechte J, Zucoloto AZ, Yu IL, Doig CJ, Dunbar MJ, McCoy KD (2023). Dysbiosis of a microbiota-immune metasystem in critical illness is associated with nosocomial infections. Nat Med.

[51] Escudero-Bautista S, Omaña-Covarrubias A, Nez-Castro AT, López-Pontigo L, Pimentel-Pérez M, Chávez-Mejía A (2024). Impact of gut microbiota on aging and frailty: a narrative review of the literature. Geriatrics (Basel).

[52] Fishbein SR, Mahmud B, Dantas G (2023). Antibiotic perturbations to the gut microbiome. Nat Rev Microbiol.

[53] Cusumano G, Flores GA, Venanzoni R, Angelini P (2025). The impact of antibiotic therapy on intestinal microbiota: dysbiosis, antibiotic resistance, and restoration strategies. Antibiotics (Basel).

[54] Bar-Yoseph H, Metcalfe-Roach A, Cirstea M, Finlay BB (2024). Microbiome changes under enteral deprivation are dynamic and dependent on intestinal location. JPEN J Parenter Enteral Nutr.

[55] Bansal S, Nguyen JP, Leligdowicz A, Zhang Y, Kain KC, Ricciuto DR (2018). Rectal and naris swabs: practical and informative samples for analyzing the microbiota of critically ill patients. MSphere.

[56] Kuo SZ, Dettmer K, Annavajhala MK, Chong DH, Uhlemann AC, Abrams JA (2021). Associations between urinary 3-indoxyl sulfate, a gut microbiome-derived biomarker, and patient outcomes after intensive care unit admission. J Crit Care.

[57] Chernevskaya E, Beloborodova N, Klimenko N, Pautova A, Shilkin D, Gusarov V (2020). Serum and fecal profiles of aromatic microbial metabolites reflect gut microbiota disruption in critically ill patients: a prospective observational pilot study. Crit Care.

[58] Yeh A, Rogers MB, Firek B, Neal MD, Zuckerbraun BS, Morowitz MJ (2016). Dysbiosis across multiple body sites in critically ill adult surgical patients. Shock.

